# Prognostic effect of liver metastasis in lung cancer patients with distant metastasis

**DOI:** 10.18632/oncotarget.10644

**Published:** 2016-07-18

**Authors:** Yijiu Ren, Chenyang Dai, Hui Zheng, Fangyu Zhou, Yunlang She, Gening Jiang, Ke Fei, Ping Yang, Dong Xie, Chang Chen

**Affiliations:** ^1^ Department of Thoracic Surgery, Shanghai Pulmonary Hospital, Tongji University School of Medicine, Shanghai, People's Republic of China; ^2^ Division of Epidemiology, Department of Health Sciences Research, Mayo Clinic, Rochester, MN, USA

**Keywords:** lung cancer, distant metastasis, prognosis, surveillance epidemiology, end-results database

## Abstract

Because the need of clinical prognostic evaluation by specific metastatic organ, we aim to analyze the prognostic factors in lung cancer patients with M1b disease with Surveillance Epidemiology and End-Results database (SEER). This retrospective study evaluated lung cancer patients of adenocarcinoma (AD), squamous cell carcinoma (SQCC), and small cell lung cancer (SCLC) selected from SEER. We provided the prognostic correlates of overall survival (OS) and lung cancer-specific survival (LCSS) in this population. 23,679 eligible patients were included. Bone was the most common metastatic site in AD (63.1%) and SQCC (61.1%), while liver was the most prevalent site (61.9%) in SCLC. Single site metastasis was significantly associated with better outcome compared to multiple sites metastases in all patients. Among patients with single site metastasis, OS and LCSS were longer for AD and SCLC if involving brain or bone, with median survival time of 5 to 7 months, comparing to 3 months if invloving liver (all *p*-values < 0.001). Similarly, among patients with multiple metastases, better outcomes were observed in AD patients (4 vs 3 months; OS and LCSS, *p* < 0.001) and SCLC patients (6 vs 4 months; OS, *p* = 0.017; LCSS, *p* = 0.023) without liver metastasis compared to those with liver metastasis. In conclusion, we estimated multiple survival outcomes by histology of primary tumor and sites of metastasis. Liver metastasis is found to be the worst prognostic factor for AD and SCLC patients with distant metastasis. More in-depth research is warranted to identify patients who are prone to develop distance metastasis, especially to liver.

## INTRODUCTION

Lung cancer is the second most commonly diagnosed cancer among men and women in the United States [1]. Approximately 40% of newly diagnosed patients present with metastatic disease [2]. In the seventh edition of the tumor, node and metastasis classification of lung cancer, distant metastasis was categorized as M1b, with only 1% of patients being alive at 5 years [3]. As reported, 25%–40% of non-small cell lung cancer patients developed brain metastases [4], while 10% of SCLC patients had brain metastases at time of diagnosis [5]. Lung cancer other than SCLC had almost the same frequency of liver metastasis (2.9–4.1%), whereas in SCLC patients 17.5%-20.3% developed liver metastasis [6, 7].

Identifying factors with the prediction value of long-term survival may contribute to managing disease [8]. Morgensztern et al. found better survival rates of AD compared to SQCC in stage IV lung cancer patients [9]. Waqar et al. suggested that brain irradiation is 67% more likely to be used in AD compared to SQCC [10]. Eberhardt et al. reported that single metastatic lesions in the adrenals showed significantly poor prognosis, which could not be confirmed in all patient groups analyzed [11]. However, previous studies could not substantiate any organ system with a significantly different prognosis compared to others.

Hence, the objective of the present study was to evaluate the prognostic correlates of overall survival (OS) and lung cancer-specific survival (LCSS) in lung cancer patients with distant metastasis by using the Surveillance, Epidemiology, and End Results (SEER) database.

## RESULTS

### Study population

This study included 23,679 M1b patients who met the inclusion criteria. The median follow-up time was 3 months (range, 0–35). Median age at diagnosis was 66 years (range, 16–112). The histologic distribution included 13,394 patients (56.6%) with AD, 3,826 patients (16.2%) with SQCC, and 6,459 patients (27.3%) with SCLC.

### Metastasis pattern

Table 1 shows the distribution of specific metastatic sites for all M1b patients. Bone was the most common metastatic site for M1b patients with AD (63.1%) and SQCC (61.1%, *p <* 0.001) while SCLC patients had a higher incidence rate of liver (61.9%) compared to other metastasis (*p <* 0.001).

**Table 1:** Clinical features and metastasis sites

Features	Bone metastasis (%)		*P* value	Brain metastasis (%)		*P* value	Liver metastasis (%)		*P* value
	No	Yes		No	Yes		No	Yes	
Age			0.357			**< 0.001**			**< 0.001**
≤ 65	4841 (48.4)	6539 (47.8)		6293 (43.8)	5087 (54.7)		7343 (49.3)	4037 (45.9)	
> 65	5159 (51.6)	7140 (52.2)		8081 (56.2)	4218 (45.3)		7544 (50.7)	4755 (54.1)	
Sex			**< 0.001**			**< 0.001**			0.525
Female	4715 (47.2)	5850 (42.8)		6099 (42.4)	4466 (48.0)		6666 (44.8)	3899 (44.3)	
Male	5285 (52.8)	7829 (57.2)		8275 (57.6)	4839 (52.0)		8221 (55.2)	4893 (55.7)	
Race			**< 0.001**			**< 0.001**			**< 0.001**
White	8074 (80.7)	11049 (80.8)		11823 (82.3)	7300 (78.5)		11814 (79.4)	7309 (83.1)	
Black	1277 (12.8)	1566 (11.4)		1624 (11.3)	1219 (13.1)		1900 (12.8)	943 (10.7)	
Other	649 (6.5)	1064 (7.8)		927 (6.4)	786 (8.4)		1173 (7.9)	540 (6.1)	
Marital status			**< 0.001**			0.084			0.256
Unmarried	4592 (45.9)	5852 (42.8)		6328 (44.0)	4116 (44.2)		6508 (43.7)	3936 (44.8)	
Married	4991 (49.9)	7221 (52.8)		7391 (51.4)	4821 (51.8)		7724 (51.9)	4488 (51.0)	
Unknown	417 (4.2)	606 (4.4)		655 (4.6)	368 (4.0)		655 (4.4)	368 (4.2)	
Location			0.075			**< 0.001**			**< 0.001**
Bronchus	634 (6.3)	767 (5.6)		923 (6.4)	478 (5.1)		717 (4.8)	684 (7.8)	
Lobe	7538 (75.4)	10359 (75.7)		10571 (73.5)	7326 (78.7)		11681 (78.5)	6216 (70.7)	
Overlap	111 (1.1)	136 (1.0)		155 (1.1)	92 (1.0)		127 (0.9)	120 (1.4)	
Unknown	1717 (17.2)	2417 (17.7)		2725 (19.0)	1409 (15.1)		2362 (15.9)	1772 (20.2)	
T stage			**< 0.001**			**< 0.001**			**< 0.001**
T1	1131 (11.3)	1407 (10.3)		1474 (10.3)	1064 (11.4)		1778 (11.9)	760 (8.6)	
T2	2465 (24.7)	3095 (22.6)		3277 (22.8)	2283 (24.5)		3677 (24.7)	1883 (21.4)	
T3	2084 (20.8)	2964 (21.7)		3082 (21.4)	1966 (21.1)		3178 (21.3)	1870 (21.3)	
T4	2860 (28.6)	4298 (31.4)		4321 (30.1)	2837 (30.5)		4318 (29.0)	2840 (32.3)	
TX	1460 (14.6)	1915 (14.0)		2220 (15.4)	1155 (12.4)		1936 (13.0)	1439 (16.4)	
N stage			**< 0.001**			0.003			**< 0.001**
N0	2050 (20.5)	2526 (18.5)		2733 (19.0)	1843 (19.8)		3281 (22.0)	1295 (14.7)	
N1	801 (8.0)	1031 (7.5)		1087 (7.6)	745 (8.0)		1230 (8.3)	602 (6.8)	
N2	4826 (48.3)	6435 (47.0)		6865 (47.8)	4396 (47.2)		6733 (45.2)	4528 (51.5)	
N3	1692 (16.9)	2845 (20.8)		2731 (19.0)	1806 (19.4)		2764 (18.6)	1773 (20.2)	
NX	631 (6.3)	842 (6.2)		958 (6.7)	515 (5.5)		879 (5.9)	594 (6.8)	
With M1a			0.022			**< 0.001**			**< 0.001**
No	9746 (97.5)	13262 (97.0)		13914 (96.8)	9094 (97.7)		14399 (96.7)	8609 (97.9)	
Yes	254 (2.5)	417 (3.0)		460 (3.2)	211 (2.3)		488 (3.3)	183 (2.1)	
Unknown	22 (0.2)	15 (0.1)		23 (0.2)	14 (0.2)		23 (0.2)	14 (0.2)	
Radiotherapy			**< 0.001**			**< 0.001**			**< 0.001**
No	4573 (45.7)	6646 (48.6)		9013 (62.7)	2206 (23.7)		5445 (36.6)	5774 (65.7)	
Yes	5266 (52.7)	6874 (50.3)		5173 (36.0)	6967 (74.9)		9236 (62.0)	2904 (33.0)	
Unknown	161 (1.6)	159 (1.2)		188 (1.3)	132 (1.4)		206 (1.4)	114 (1.3)	

AD: adenocarcinoma, SQCC: squamous cell carcinoma, SCC: small cell carcinoma, M1a: Separate tumor nodules in a contralateral lobe or tumor with pleural nodules or malignant pleural dissemination; Bold values corresponds to the comparisons with *P* < 0.001.

**Figure 1:**
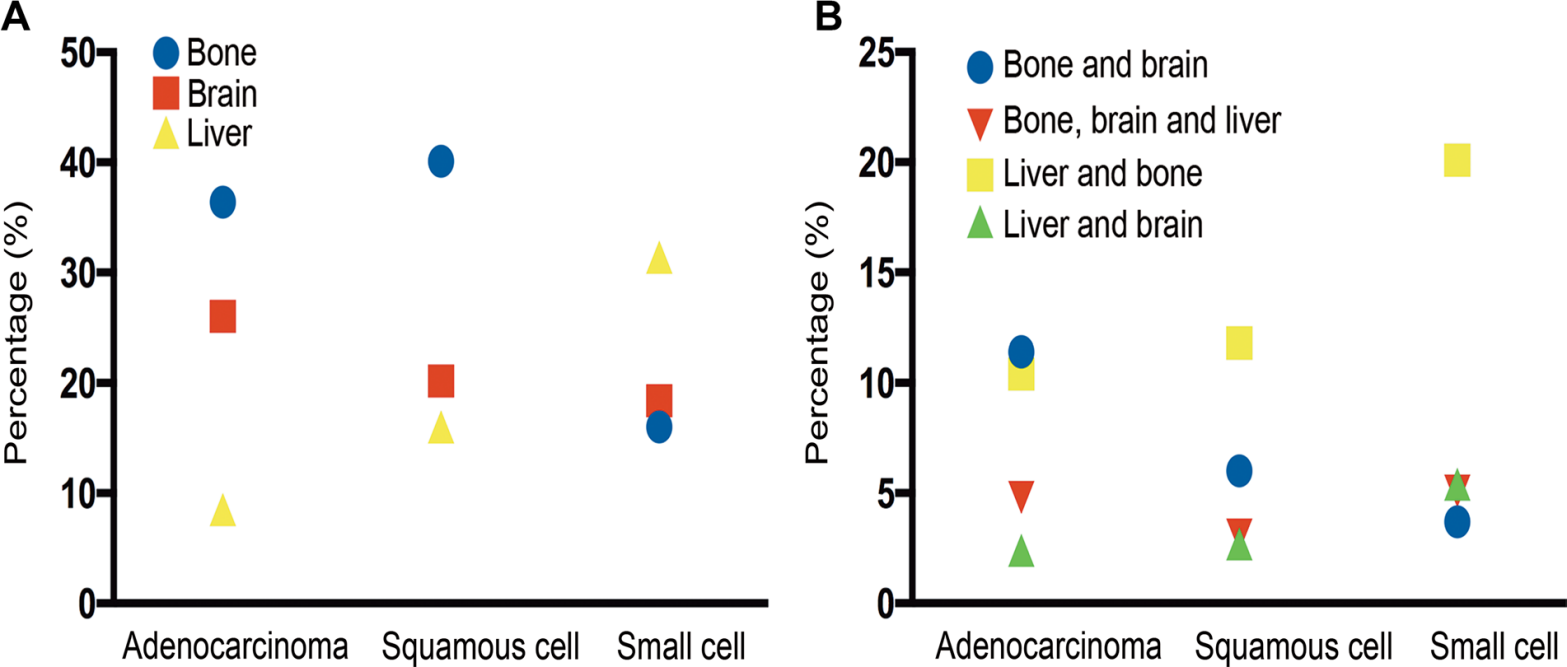
(**A**) Metastatic rate in lung cancer patients with single metastatic site. (**B**) Metastatic rate in lung cancer patients with multiple metastatic sites.

Supplementary Table S1 and Figure 1 summarizes all the combination of these three sites of metastasis. Considering single site metastasis, about 36.4% of AD patients and 40.1% of SQCC patients had only bone metastasis while 31.4% of SCLC patients had only liver metastasis. The most common two-site metastasis combination was bone and brain for AD patients (11.4%), bone and liver for SQCC patients (11.8%), and bone and liver for SCLC patients (20.1%). We also found that SCLC patients were most likely to have multiple sites metastases, especially liver combined with other sites.

### Survival

The median survival time (MST) and 2-year survival rate of AD patients were 5 months and 12.3% (OS), and 5 months and 13.8% (LCCS). The median survival time and 2-year survival rate of SQCC patients were 3 months and 4.1% (OS), and 3 months and 5.1% (LCCS), respectively. The median survival time and 2-year survival rate of SCLC patients were 5 months and 3.9% (OS), and 6 months and 4.7% (LCCS), respectively. Kaplan-Meier analysis showed that AD was significantly associated with better OS and LCSS compared to SQCC patients (OS and LCSS, *p <* 0.001) (Supplementary Figure S1). Multivariable analyses also indicated that SQCC was significantly associated with decreased OS (HR, 1.303; 95% CI, 1.251-1.358; *p <* 0.001) and LCSS (HR, 1.309; 95% CI, 1.254-1.365; *p <* 0.001) compared to AD (Table 2).

**Table 2:** Multivariate analysis of overall survival and lung cancer-specific survival in lung cancer patients with distant metastasis

Features	Overall survival		Lung cancer-specific survival	
	Hazard Ratios (95% CI)	*P* Value	Hazard Ratios (95% CI)	*P* Value
Age				
≤ 65 y	1.00 (Reference)		1.00 (Reference)	
> 65 y	1.347 (1.307–1.389)	**< 0.001**	1.341 (1.300–1.383)	**< 0.001**
Gender				
Female	1.00 (Reference)		1.00 (Reference)	
Male	1.197 (1.161–1.234)	**< 0.001**	1.182 (1.145–1.219)	**< 0.001**
Married				
No	1.00 (Reference)		1.00 (Reference)	
Yes	0.815 (0.790–0.840)	**< 0.001**	0.824 (0.798–0.851)	**< 0.001**
Unknown	0.912 (0.847–0.982)	0.015	0.896 (0.830–0.968)	0.005
Race				
White	1.00 (Reference)		1.00 (Reference)	
Black	0.988 (0.944–1.035)	0.611	0.976 (0.931–1.024)	0.320
Other	0.719 (0.675–0.764)	**< 0.001**	0.707 (0.663–0.754)	**< 0.001**
Location				
Main bronchus	1.00 (Reference)		1.00 (Reference)	
Single Lobe	0.938 (0.881–0.999)	0.046	0.944 (0.885–1.007)	0.082
Overlap	0.960 (0.822–1.122)	0.610	0.946 (0.805–1.111)	0.497
Unknown	1.095 (1.020–1.174)	0.012	1.095 (1.019–1.177)	0.014
T status				
T1	1.00 (Reference)		1.00 (Reference)	
T2	1.170 (1.106–1.238)	**< 0.001**	1.171 (1.105–1.241)	**< 0.001**
T3	1.269 (1.198–1.344)	**< 0.001**	1.270 (1.197–1.347)	**< 0.001**
T4	1.260 (1.192–1.331)	**< 0.001**	1.265 (1.195–1.338)	**< 0.001**
TX	1.287 (1.208–1.371)	**< 0.001**	1.294 (1.212–1.380)	**< 0.001**
Lymph node status				
N0	1.00 (Reference)		1.00 (Reference)	
N1	1.045 (0.981–1.114)	0.169	1.055 (0.989–1.126)	0.107
N2	1.138 (1.092–1.186)	**< 0.001**	1.139 (1.091–1.188)	**< 0.001**
N3	1.107 (1.054–1.163)	**< 0.001**	1.111 (1.056–1.168)	**< 0.001**
NX	1.176 (1.098–1.260)	**< 0.001**	1.173 (1.092–1.259)	**< 0.001**
With M1a				
No	1.00 (Reference)		1.00 (Reference)	
Yes	1.001 (0.918–1.092)	0.980	0.981 (0.896–1.073)	0.673
Distant metastasis				
Single site	1.00 (Reference)		1.00 (Reference)	
Multiple sites	1.303 (1.261–1.346)	**< 0.001**	1.321 (1.278–1.366)	**< 0.001**
Histology				
Adenocarcinoma	1.00 (Reference)		1.00 (Reference)	
Squamous cellcarcinoma	1.303 (1.251–1.358)	**< 0.001**	1.309 (1.254–1.365)	**< 0.001**
Small cell lung cancer	1.044 (1.008–1.082)	0.017	1.045 (1.007–1.083)	0.019
Radiotherapy				
Yes	1.00 (Reference)		1.00 (Reference)	
No	1.772 (1.594–1.971)	**< 0.001**	0.752 (0.729–0.776)	**< 0.001**
Unknown	1.265 (1.002–1.598)	0.048	0.814 (0.706–0.939)	0.005

M1a: Separate tumor nodules in a contralateral lobe or tumor with pleural nodules or malignant pleural dissemination; Bold values corresponds to the comparisons with *P* < 0.001.

**Figure 2:**
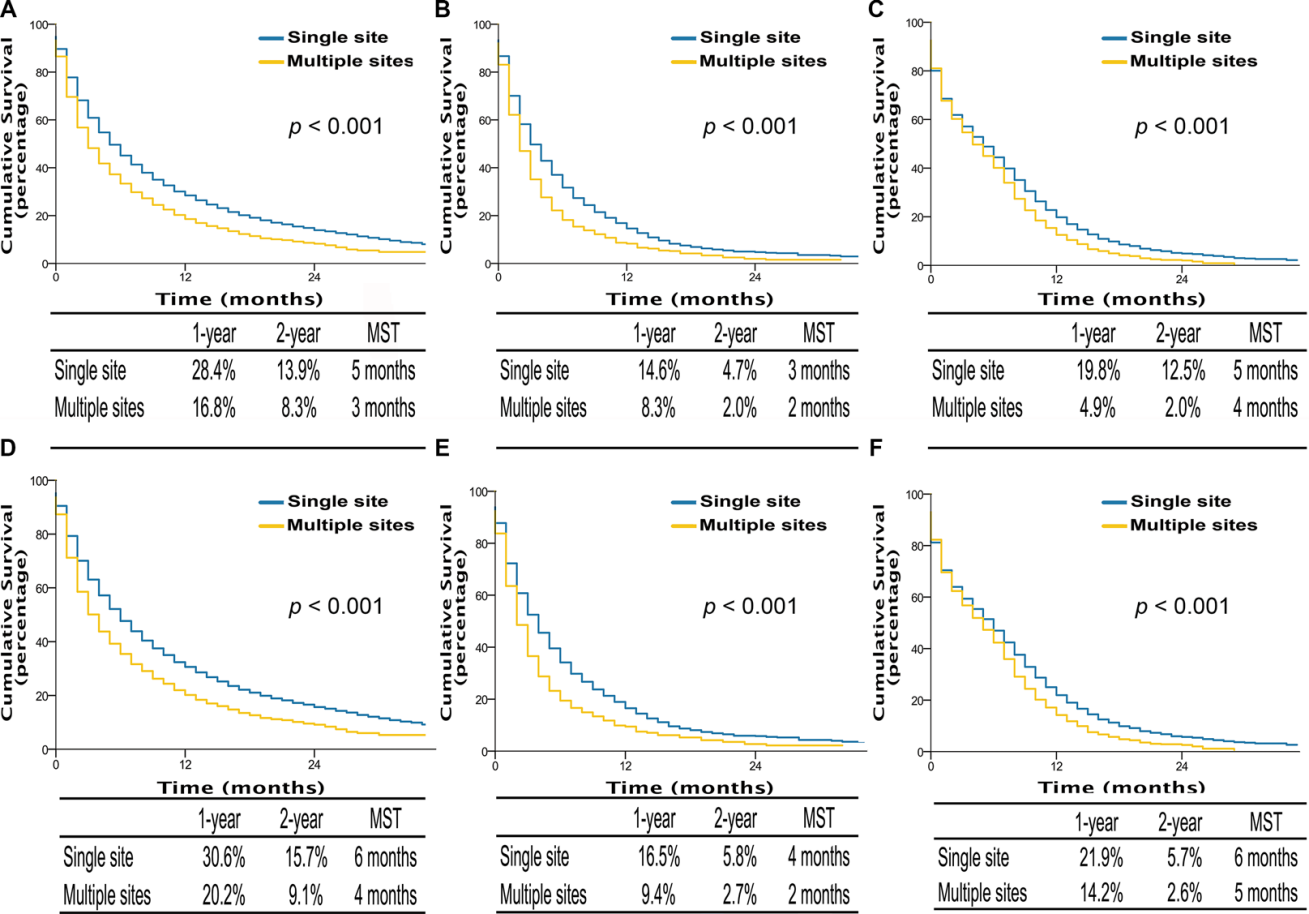
Overall survival in lung cancer patients with adenocarcinoma, squamous cell carcinoma, and small cell lung cancer with single site metastasis or multiple sites (A, B, and C); lung cancer-specific survival in lung cancer patients of adenocarcinoma, squamous cell carcinoma, and small cell lung cancer with single site metastasis or multiple sites (D, E, and F) MST, The median survival time.

Kaplan-Meier analysis also showed that single site metastasis was significantly associated with better OS and LCSS compared to multiple sites metastasis in AD patients (OS, MST, 5 vs 3 months, *p <* 0.001; LCSS, MST, 6 vs 4 months, *p <* 0.001), SQCC patients (OS, MST, 3 vs 2 months, *p <* 0.001; LCSS, MST, 4 vs 2 months, *p <* 0.001), and SCLC patients (OS, MST, 5 vs 4 months, *p <* 0.001; LCSS, MST, 6 vs 5 months, *p <* 0.001) (Figure 2). Similarly, multivariable analyses suggested that decreased OS and LCSS were associated with multiple sites metastasis among AD patients, SQCC patients, and SCLC patients compared to single site metastasis (Supplementary Table S2–S4).

**Figure 3:**
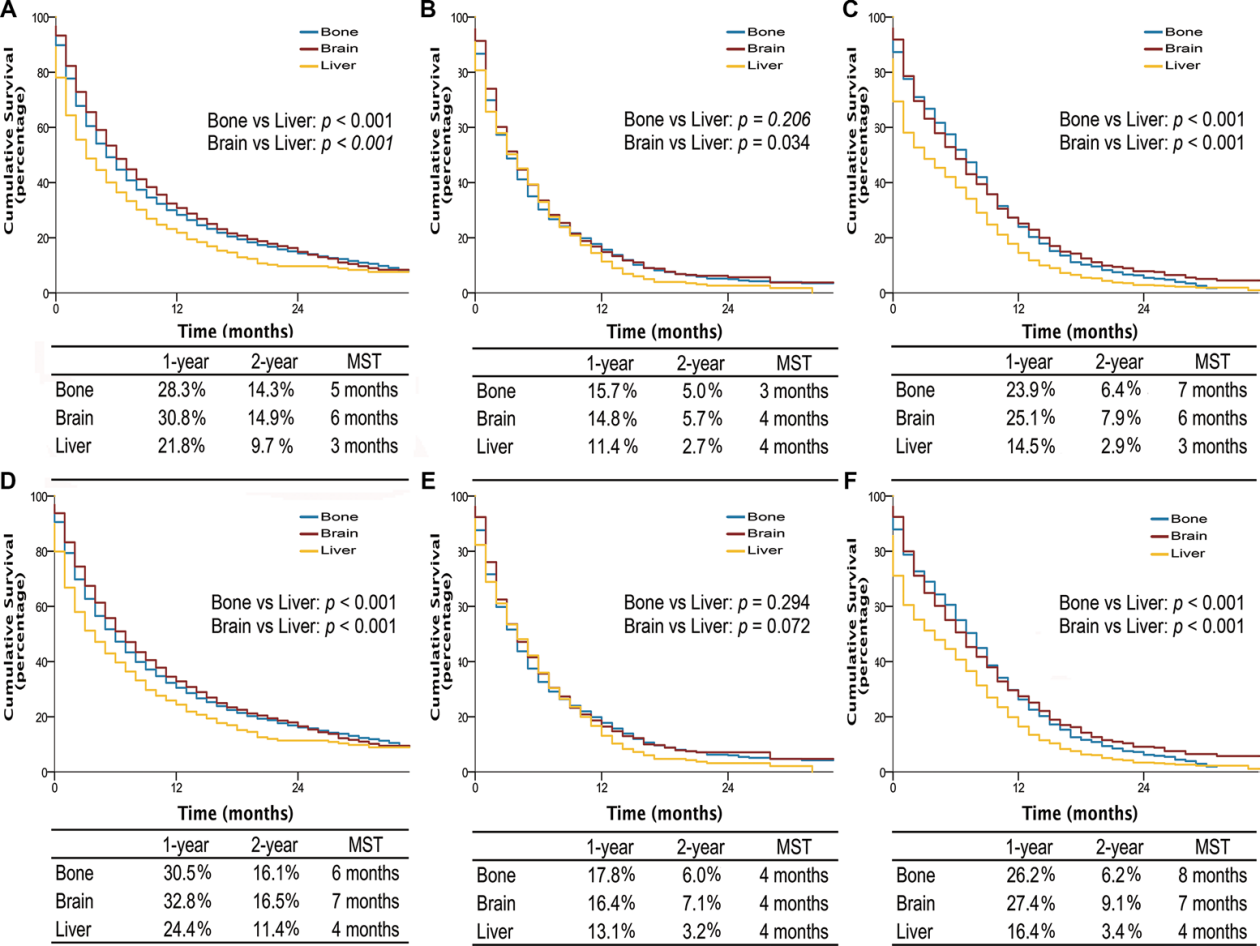
Overall survival in lung cancer patients with adenocarcinoma, squamous cell carcinoma, and small cell lung cancer with single site metastasis of different organ (A, B, and C); lung cancer-specific survival in lung cancer patients of adenocarcinoma, squamous cell carcinoma, and small cell lung cancer with single site metastasis of different organ (D, E, and F) MST, The median survival time.

**Figure 4:**
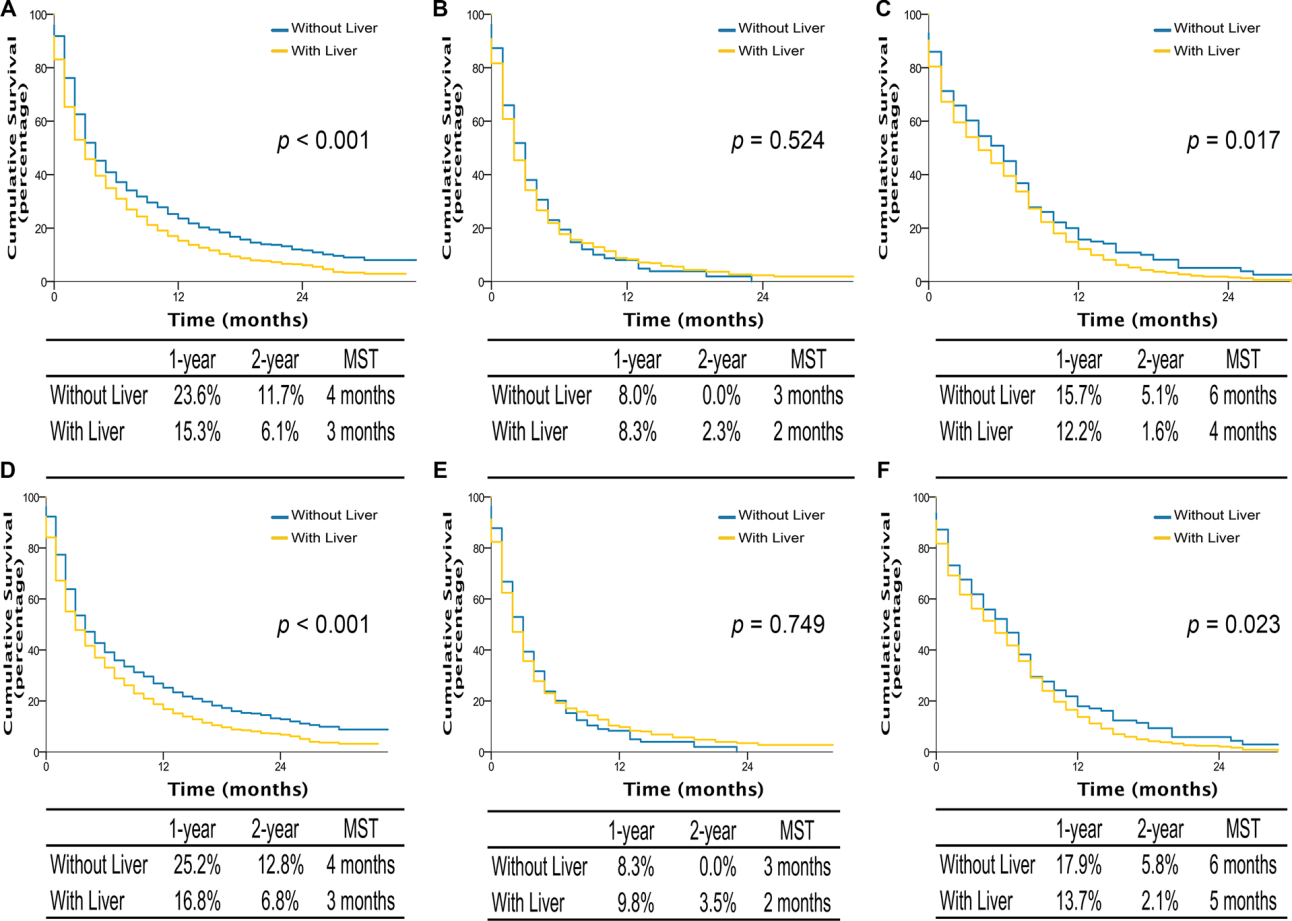
Overall survival in lung cancer patients with adenocarcinoma, squamous cell carcinoma, and small cell lung cancer with multiple sites metastases of different organ combination (A, B, and C); lung cancer-specific survival in lung cancer patients of adenocarcinoma, squamous cell carcinoma, and small cell lung cancer with multiple sites metastases of different organ combination (D, E, and F) With liver, multiple sites including liver metastasis. Without liver, multiple sites not including liver metastasis. MST, The median survival time.

Interestingly, among patients with single site metastasis, OS and LCSS were longer for AD and SCLC if involving brain or bone, with median survival time (MST) of 5 to 7 months, comparing to 3 months if invloving liver (all *p*-values < 0.001) (Figure 3). Similarly, among patients with multiple metastases, better OS and LCSS were observed in AD patients (MST, 4 vs 3 months; OS and LCSS, *p* < 0.001) and SCLC patients without liver metastasis (MST, 6 vs 4 months; OS, *p* = 0.017; LCSS, *p* = 0.023) compared to those with liver metastasis (Figure 4). Multivariable analyses also suggested that decreased OS and LCSS were associated with liver metastasis among AD patients, and SCLC patients (Supplementary Table S5–S8).

## DISCUSSION

The main finding of this study is that, the AD patients and SCLC patients in M1b lung cancer without liver metastasis had a better prognosis than those with liver metastasis. We (1) confirmed major difference in metastatic frequencies among lung cancer patients with AD, SQCC, and SCLC and (2) identified prognostic factors of specific combinations of metastatic sites.

Knowledge of difference in metastatic patterns may be useful in making diagnosis of metastasis and treatment decision. Regarding the metastatic patterns of different histologic types, we found that both AD and SQCC metastasize predominantly to bone. Similarly, previous studies have reported that 30–40% of patients with lung cancer developed bone metastases with median survival time of 7 months [4]. Interestingly, we found that SCLC metastasizes predominantly to liver (61.9%). The incidence of liver metastasis in patients with lung cancer has been reported to be 37–51% in autopsy [12]. Nakazawa et al. reported that among 251 SCLC patients diagnosed, 79 patients had distant metastasis, of which 51 (64.5%) of metastatic patients had liver metastasis, which also supported out results [13].

The effect of histology on survival of lung cancer patients with distant metastasis has not been well delineated. In a recent research, Morgensztern et al. evaluated the role of histology in NSCLC stage IV from 1990 to 2005 among the SEER data [9]. However, only in the time period between 2002 and 2005, it was observed increased survival for AD compared with SQCC (OS, HR, 1.033, *p* = 0.02), while there were no significant survival difference between AD and SQCC before 2002 [9]. In previous trials, AD showed increased response rates compared with other histologies, which was supported by subsequent phase III studies using gefitinib or erlotinib [14]. Among patients having pemetrexed, AD showed better outcome compared with SQCC [15]. Since TKIs has spread between 2010 and 2012, the recent differences in outcomes based on observed histology may reflect the increased activity of TKIs in AD compared to SQCC.

Our results showed that the prognosis of patients with single metastatic site was better than those with multiple sites, which is consistent with previous reports. Retrospective data have suggested prognostic differences between patients with single metastatic site and those with multiple sites. Escuín et al. reported that patients with multiple metastatic sites had poorer survival compared to those with isolated metastasis and multiple lesions (*p* = 0.024) [16]. Similarly, Eberhardt et al. reported prognostic differences for subjects with a single metastatic lesion in a single site (MST, 11 months) compared to all other patient groups with multiple metastatic lesions in a single site and with multiple lesions in multiple sites (MST, 6 months) [11]. Our findings also suggest that the prognostic differences between single metastatic site and multiple sites exist in three main histologic types of lung cancer (AD, MST 5 vs 3 months; SQCC, MST 3 vs 2 months; SCLC, MST 5 vs 4 months, *p* < 0.001). Hence, all these evidence strongly support the proposal eighth edition of the TNM classification, [11] including adding following characteristics in the next TNM classification, specifically, (a) number of metastatic lesions of each involved site, (b) diameter of individual metastatic lesions, and (c) number of involved sites.

Previous studies stated that some organ systems, such as liver, would show a significantly different prognosis compared to others (Table 3). Nakazawa et al. found that the mortality risk with liver metastasis was 2.41-fold higher than other distant metastasis (*P* < 0.001) [13]. Riihimäki et al. found that the mortality risk with liver metastasis was 1.53-fold higher than brain metastasis (*P* < 0.05) [17]. Tamura et al. found that the mortality risk with liver metastasis was 1.55-fold higher than other distant metastasis (*P* < 0.001) [18]. Similarly, we found that liver metastasis had a significantly worse prognosis compared to brain and bone metastasis in AD and SCLC patients with only one metastatic site. Additionally, in AD and SCLC patients with multiple metastatic sites, combination with liver metastasis showed worse outcome compared to other combinations without liver metastasis. Although we could not separate a single lesion from multiple lesions in liver using SEER database, liver metastasis still may be considered a negative prognostic factor for AD and SCLC patients. The current standard treatment for liver metastasis is chemotherapy and, when appropriate, local radiation therapy [8]. When a patient has one or two liver nodules with more than one year of disease-free interval from the resection of primary lung cancer, hepatic resection may be a therapeutic option [19]. Unlike bone or brain metastasis, few studies focused on liver metastasis of lung cancer. More efforts should be made to work out effective treatment strategies and consensus about liver metastasis of lung cancer.

**Table 3:** The summary of previous research articles about lung cancer metastasis

Authors	Patient numbers	Diagnosed time span	Histologic types	Main results	Drawback
Nakazawa et al.	251	1999–2010	Small cell lung cancer	Liver, bone and brain metastasis, pleural and/ or pericardial fluids were unfavorable prognostic factors	Small sample size; long and retrospective period of patients' inclusion
Riihimäki et al.	3,759	2002–2010	All histologic types	Liver and bone metastases signal poor survival, compared with nervous system metastases.	Only in Swedish population; long and retrospective period of patients' inclusion
Tamura et al.	1,542	1999–2012	Non small cell lung cancer	Liver and adrenal gland metastases were unfavorable prognostic factors	Not including small cell lung cancer.

All cited in reference.

To our knowledge, this is the first SEER based study focused on the metastatic pattern of different histologic types of primary lung cancer, considering them separate entities. However, there are obvious limitations, as outlined below. First, it is important to note that the database contains only the data collected between 2010 and 2012. Furthermore, we have only information on synchronous metastasis to bone, brain, and liver that affects significantly less patients compared to those patients who will develop metachronous metastatic lesions and other organs. These limitations may have led to an underestimation of other sites of metastasis, but as previous reports estimated, the three sites of metastasis assessed accounted for 80% of lung cancer patients [9, 11]. Moreover, because the SEER database set was not integrated for distant metastasis, interesting information, such as number of metastatic lesions, AD subtypes, SQCC subtpyes, ECOG performance status, tumor mutation, and therapy type were not included.

In conclusion, based on the SEER data, we estimated and compared multiple survival outcomes for M1b lung cancer patients by histology of primary tumor and sites of metastasis. Liver metastasis is found to be the worst prognostic factor for AD and SCLC patients with distant metastasis. More in-depth research is warranted to identify patients who are prone to develop distance metastasis, especially to liver.

## MATERIALS AND METHODS

### Study population

We hypothesize that SEER is a good database from which to analyze the distant metastasis pattern for lung cancers. So, we sent an application through a SEER custom data request and received their permission. This database only includes metastasis to the bone, brain, and liver at the time of diagnosis.

The dataset we used for analysis was “Incidence- SEER 18 Custom Data (with CS mets at DX fields), Nov 2014 Sub (2010–2012). SEER 18 Regs Custom Data (Malignant only, Nov 2014 Sub (2010–2012)) were used to identify patients who met the inclusion criteria (site = lung and bronchus, behavior = malignant, and year of diagnosis = 2010–2012) [20]. In addition, we included only patients who had (1) pathologically confirmed lung cancer with pathological types of AD, SQCC, or SCLC, (2) M1b disease only due to bone, brain, or liver metastasis (SEER code: if any of CS mets at DX-bone, brain, or liver was “1”), and (3) only one malignant primary tumor.

We collected the demographic characteristics of patients (age, gender, marriage, and race), pathological features of tumors (location, histological type, T stage, N stage, and M1a stage), and types of therapeutic management (surgical type, and radiotherapy) from SEER database. In this study, pathological types were classified as AD (SEER codes 8140, 8230, 8254, 8255, 8260, 8310, 8333, 8470, 8480, 8481, 8490 and 8550), SQCC (SEER codes 8052, 8070, 8071, 8072, 8073, 8083 and 8084), and SCLC (SEER codes 8002, 8041, 8043, 8044 and 8045). Since OS and LCSS were also included in SEER database, both of them were regarded as the outcomes of interest. Patient outcomes were obtained up to November 31, 2014. OS (SEER code: Vital status recode was “Dead” or “Alive”) was defined as the survival time from diagnose until death from any cause or until the last follow-up, and LCSS (SEER code: SEER cause-specific death classification was “Dead” or “Alive”) as the survival time from surgery until cause-specific death due to lung cancer or until the last follow-up.

### Statistical analysis

The data were presented as frequencies (percent) or median (range) deviation. The comparison of demographic, pathologic, and therapeutic features between metastasis sites was performed using unpaired *t*-test for continuous variables and Pearson χ^2^ test for categorical variables. The OS and LCSS were analyzed by using the Kaplan-Meier method and the log-rank test comparing survival in two or more groups. Multivariate Cox proportional hazard analyses were applied to adjust the potential confounders related to demographic, pathologic, and therapeutic features in the survival analysis. A two-sided *p* value < 0.05 was regarded statistically significant. All analyses were conducted using SPSS 23.0 (SPSS Inc. Chicago, IL) and bar chart were drawn using GraphPad Prism 6.0 (GraphPad Software, San Diego, CA).

## SUPPLEMENTARY MATERIALS FIGURE AND TABLES



## Abbreviations

SEER: Surveillance Epidemiology and End-Results database
AD: adenocarcinoma
SQCC: squamous cell carcinoma
SCLC: small cell lung cancer
OS: overall survival
LCSS: lung cancer-specific survival
MST: The median survival time
