# Supplementary material for: Prognostic effect of liver metastasis in lung cancer patients with distant metastasis
Source: Oncotarget. 2016 Jul 18;7(33):53245–53. doi: 10.18632/oncotarget.10644 (PMC5288182; doi:10.18632/oncotarget.10644)
Supplement: Supplementary file 1 [file oncotarget-07-53245-s001.pdf]

Prognostic effect of liver metastasis in lung cancer patients with distant metastasis

Supplementary Materials

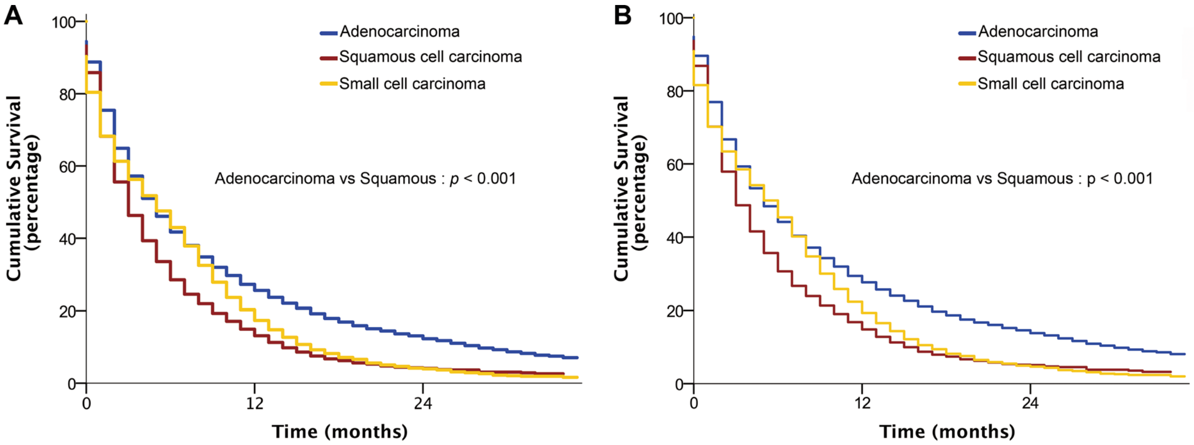

A

	1-year	2-year
Adenocarcinoma	25.6%	12.3%
Squamous cell carcinoma	13.1%	4.1%
Small cell carcinoma	17.3%	3.9%

B

	1-year	2-year
Adenocarcinoma	27.6%	13.8%
Squamous cell carcinoma	14.8%	5.1%
Small cell carcinoma	19.3%	4.7%

Supplementary Figure S1: (**A**) Overall survival in lung cancer patients of adenocarcinoma, squamous cell carcinoma, and small cell carcinoma with distant metastasis; (**B**) lung cancer-specific survival in lung cancer patients of adenocarcinoma, squamous cell carcinoma, and small cell carcinoma with distant metastasis.

Supplementary Table S1: Frequencies of combination metastasis

Features	Adenocarcinoma		Squamous cell carcinoma		Small cell lung cancer		*P* value
	Number	(%)	Number	(%)	Number	(%)	
Single site							
Only bone	4877	36.4	1536	40.1	1032	16.0	**< 0.001**
Only brain	3479	26.0	774	20.2	1188	18.4	**< 0.001**
Only liver	1145	8.5	611	16.0	2025	31.4	**< 0.001**
Multiple sites							
Bone and brain	1530	11.4	229	6.0	242	3.7	**< 0.001**
Bone and liver	1399	10.4	453	11.8	1296	20.1	**< 0.001**
Brain and liver	325	2.4	105	2.7	348	5.4	**< 0.001**
Bone, brain and liver	639	4.8	118	3.1	328	5.1	**< 0.001**

Bold values corresponds to the comparisons with $P < 0.001$.

Supplementary Table S2: Multivariate analysis of overall survival and lung cancer-specific survival in adenocarcinoma patients with distant metastasis

Features	Overall survival		Lung cancer-specific survival	
	**Hazard Ratios (95% CI)**	***P* Value**	**Hazard Ratios (95% CI)**	***P* Value**
Age				
≤ 65 y	1.00 (Reference)		1.00 (Reference)	
> 65 y	1.368 (1.313–1.425)	**< 0.001**	1.364 (1.308–1.423)	**< 0.001**
Gender				
Female	1.00 (Reference)		1.00 (Reference)	
Male	1.284 (1.232–1.337)	**< 0.001**	1.266 (1.214–1.320)	**< 0.001**
Married				
No	1.00 (Reference)		1.00 (Reference)	
Yes	0.798 (0.765–0.832)	**< 0.001**	0.806 (0.772–0.842)	**< 0.001**
Unknown	0.914 (0.829–1.007)	0.070	0.887 (0.801–0.982)	0.020
Race				
White	1.00 (Reference)		1.00 (Reference)	
Black	0.992 (0.934–1.054)	0.804	0.984 (0.924–1.047)	0.613
Other	0.662 (0.614–0.713)	**< 0.001**	0.656 (0.607–0.708)	**< 0.001**
Location				
Main bronchus	1.00 (Reference)		1.00 (Reference)	
Single Lobe	0.798 (0.717–0.889)	**< 0.001**	0.811 (0.726–0.906)	**< 0.001**
Overlap	0.812 (0.641–1.028)	0.084	0.832 (0.653–1.060)	0.137
Unknown	0.972 (0.866–1.091)	0.627	0.978 (0.868–1.102)	0.713
T status				
T1	1.00 (Reference)		1.00 (Reference)	
T2	1.171 (1.089–1.259)	**< 0.001**	1.182 (1.097–1.274)	**< 0.001**
T3	1.291 (1.199–1.390)	**< 0.001**	1.299 (1.204–1.402)	**< 0.001**
T4	1.307 (1.217–1.403)	**< 0.001**	1.323 (1.230–1.423)	**< 0.001**
TX	1.325 (1.220–1.439)	**< 0.001**	1.333 (1.224–1.451)	**< 0.001**
Lymph node status				
N0	1.00 (Reference)		1.00 (Reference)	
N1	1.019 (0.936–1.110)	0.664	1.026 (0.940–1.121)	0.565
N2	1.159 (1.098–1.223)	**< 0.001**	1.164 (1.101–1.230)	**< 0.001**
N3	1.162 (1.098–1.239)	**< 0.001**	1.169 (1.095–1.249)	**< 0.001**
NX	1.178 (1.076–1.288)	**< 0.001**	1.169 (1.065–1.283)	**< 0.001**
With M1a				
No	1.00 (Reference)		1.00 (Reference)	
Yes	0.993 (0.888–1.111)	0.906	0.977 (0.870–1.098)	0.699
Distant metastasis				
Single site	1.00 (Reference)		1.00 (Reference)	
Multiple sites	1.375 (1.315–1.437)	**< 0.001**	1.392 (1.331–1.457)	**< 0.001**
Radiotherapy				
No	1.00 (Reference)		1.00 (Reference)	
Yes	0.791 (0.759–0.824)	**< 0.001**	0.803 (0.769–0.838)	**< 0.001**
Unknown	0.813 (0.663–0.997)	0.046	0.807 (0.653–0.997)	0.047

M1a: Separate tumor nodules in a contralateral lobe or tumor with pleural nodules or malignant pleural dissemination; Bold values corresponds to the comparisons with $P < 0.001$.

Supplementary Table S3: Multivariate analysis of overall survival and lung cancer-specific survival in squamous cell carcinoma patients with distant metastasis

Features	Overall survival		Lung cancer-specific survival	
	Hazard Ratios (95% CI)	*P* Value	Hazard Ratios (95% CI)	*P* Value
Age				
≤ 65 y	1.00 (Reference)		1.00 (Reference)	
> 65 y	1.254 (1.156–1.341)	**< 0.001**	1.225 (1.135–1.322)	**< 0.001**
Gender				
Female	1.00 (Reference)		1.00 (Reference)	
Male	1.047 (0.971–1.130)	0.232	1.048 (0.969–1.134)	0.239
Married				
No	1.00 (Reference)		1.00 (Reference)	
Yes	0.894 (0.831–0.963)	0.003	0.911 (0.844–0.983)	0.016
Unknown	0.980 (0.826–1.164)	0.820	0.964 (0.806–1.153)	0.691
Race				
White	1.00 (Reference)		1.00 (Reference)	
Black	0.966 (0.871–1.071)	0.512	0.935 (0.839–1.041)	0.219
Other	0.919 (0.783–1.077)	0.296	0.930 (0.790–1.094)	0.382
Location				
Main bronchus	1.00 (Reference)		1.00 (Reference)	
Single Lobe	0.916 (0.793–1.058)	0.231	0.925 (0.798–1.073)	0.305
Overlap	1.055 (0.696–1.600)	0.801	0.983 (0.632–1.531)	0.691
Unknown	0.988 (0.832–1.173)	0.889	1.005 (0.841–1.200)	0.960
T status				
T1	1.00 (Reference)		1.00 (Reference)	
T2	1.167 (0.992–1.373)	0.062	1.135 (0.962–1.339)	0.135
T3	1.259 (1.071–1.481)	0.005	1.237 (1.049–1.459)	0.012
T4	1.278 (1.089–1.500)	0.003	1.261 (1.071–1.484)	0.005
TX	1.276 (1.061–1.535)	0.010	1.219 (1.009–1.473)	0.040
Lymph node status				
N0	1.00 (Reference)		1.00 (Reference)	
N1	1.165 (1.017–1.335)	0.028	1.186 (1.031–1.363)	0.017
N2	1.135 (1.034–1.245)	0.008	1.134 (1.031–1.248)	0.010
N3	1.059 (0.941–1.192)	0.339	1.048 (0.927–1.184)	0.454
NX	1.149 (0.965–1.368)	0.119	1.165 (0.974–1.395)	0.095
With M1a				
No	1.00 (Reference)		1.00 (Reference)	
Yes	1.016 (0.841–1.227)	0.870	1.005 (0.826–1.222)	0.962
Distant metastasis				
Single site	1.00 (Reference)		1.00 (Reference)	
Multiple sites	1.346 (1.238–1.462)	**< 0.001**	1.380 (1.268–1.503)	**< 0.001**
Unknown	1.161 (0.537–2.512)	0.705	1.069 (0.466–2.453)	0.875
Radiotherapy				
No	1.00 (Reference)		1.00 (Reference)	
Yes	0.738 (0.687–0.793)	**< 0.001**	0.762 (0.708–0.821)	**< 0.001**
Unknown	0.900 (0.656–1.235)	0.514	0.896 (0.643–1.247)	0.514

M1a: Separate tumor nodules in a contralateral lobe or tumor with pleural nodules or malignant pleural dissemination; Bold values corresponds to the comparisons with $P < 0.001$.

Supplementary Table S4: Multivariate analysis of overall survival and lung cancer-specific survival in small cell lung cancer patients with distant metastasis

Features	Overall survival		Lung cancer-specific survival	
	Hazard Ratios (95% CI)	*P* Value	Hazard Ratios (95% CI)	*P* Value
Age				
≤ 65 y	1.00 (Reference)		1.00 (Reference)	
> 65 y	1.360 (1.286–1.440)	**< 0.001**	1.359 (1.282–1.440)	**< 0.001**
Gender				
Female	1.00 (Reference)		1.00 (Reference)	
Male	1.117 (1.055–1.182)	**< 0.001**	1.098 (1.036–1.164)	0.002
Married				
No	1.00 (Reference)		1.00 (Reference)	
Yes	0.799 (0.754–0.846)	**< 0.001**	0.806 (0.760–0.856)	**< 0.001**
Unknown	0.845 (0.725–0.985)	0.032	0.860 (0.734–1.006)	0.059
Race				
White	1.00 (Reference)		1.00 (Reference)	
Black	0.982 (0.889–1.085)	0.724	0.981 (0.886–1.087)	0.720
Other	0.933 (0.798–1.090)	0.383	0.866 (0.733–1.022)	0.089
Location				
Main bronchus	1.00 (Reference)		1.00 (Reference)	
Single Lobe	1.074 (0.980–1.177)	0.128	1.073 (0.977–1.180)	0.141
Overlap	1.051 (0.827–1.337)	0.683	1.010 (0.785–1.299)	0.940
Unknown	1.202 (1.079–1.338)	0.001	1.192 (1.067–1.331)	0.002
T status				
T1	1.00 (Reference)		1.00 (Reference)	
T2	1.186 (1.062–1.324)	0.002	1.180 (1.054–1.321)	0.004
T3	1.251 (1.117–1.402)	**< 0.001**	1.245 (1.107–1.399)	**< 0.001**
T4	1.192 (1.072–1.326)	0.001	1.184 (1.062–1.320)	0.002
TX	1.205 (1.072–1.355)	0.002	1.229 (1.090–1.387)	0.001
Lymph node status				
N0	1.00 (Reference)		1.00 (Reference)	
N1	0.951 (0.833–1.086)	0.459	0.951 (0.830–1.090)	0.471
N2	1.049 (0.960–1.146)	0.289	1.038 (0.948–1.137)	0.420
N3	0.990 (0.894–1.096)	0.841	0.988 (0.890–1.096)	0.815
NX	1.187 (1.034–1.362)	0.015	1.184 (1.028–1.364)	0.019
With M1a				
No	1.00 (Reference)		1.00 (Reference)	
Yes	1.032 (0.843–1.263)	0.760	0.987 (0.798–1.222)	0.960
Distant metastasis				
Single site	1.00 (Reference)		1.00 (Reference)	
Multiple sites	1.197 (1.129–1.269)	**< 0.001**	1.210 (1.139–1.285)	**< 0.001**
Radiotherapy				
No	1.00 (Reference)		1.00 (Reference)	
Yes	0.628 (0.593–0.667)	**< 0.001**	0.631 (0.594–0.671)	**< 0.001**
Unknown	0.773 (0.607–0.985)	0.037	0.787 (0.614–1.008)	0.057

M1a: Separate tumor nodules in a contralateral lobe or tumor with pleural nodules or malignant pleural dissemination; Bold values corresponds to the comparisons with $P < 0.001$.

Supplementary Table S5: Multivariate analysis of overall survival and lung cancer-specific survival in adenocarcinoma patients with single site of distant metastasis

Features	Overall survival		Lung cancer-specific survival	
	Hazard Ratios (95% CI)	*P* Value	Hazard Ratios (95% CI)	*P* Value
Age				
≤ 65 y	1.00 (Reference)		1.00 (Reference)	
> 65 y	< 0.001	0.026	< 0.001	0.031
Gender				
Female	1.00 (Reference)		1.00 (Reference)	
Male	1.190 (1.132–1.250)	**< 0.001**	1.166 (1.107–1.227)	**< 0.001**
Married				
No	1.00 (Reference)		1.00 (Reference)	
Yes	0.984 (0.935–1.036)	0.550	0.993 (0.941–1.047)	0.786
Unknown	1.007 (0.897–1.132)	0.901	0.992 (0.878–1.120)	0.895
Race				
White	1.00 (Reference)		1.00 (Reference)	
Black	1.230 (1.144–1.322)	**< 0.001**	1.226 (1.137–1.321)	**< 0.001**
Other	0.842 (0.767–0.924)	**< 0.001**	0.834 (0.757–0.918)	**< 0.001**
Location				
Main bronchus	1.00 (Reference)		1.00 (Reference)	
Single Lobe	0.908 (0.794–1.040)	0.164	0.917 (0.797–1.054)	0.221
Overlap	0.999 (0.741–1.347)	0.995	1.051 (0.777–1.423)	0.746
Unknown	1.044 (0.902–1.207)	0.565	1.040 (0.895–1.209)	0.608
T status				
T1	1.00 (Reference)		1.00 (Reference)	
T2	1.051 (0.965–1.144)	0.253	1.051 (0.963–1.148)	0.262
T3	1.089 (0.999–1.188)	0.054	1.089 (0.996–1.191)	0.063
T4	1.131 (1.040–1.230)	0.004	1.143 (1.049–1.246)	0.002
TX	1.079 (0.978–1.190)	0.128	1.075 (0.971–1.190)	0.162
Lymph node status				
N0	1.00 (Reference)		1.00 (Reference)	
N1	1.032 (0.934–1.140)	0.531		
N2	1.164 (1.094–1.240)	**< 0.001**	1.172 (1.098–1.250)	**< 0.001**
N3	1.311 (1.214–1.416)	**< 0.001**	1.326 (1.225–1.435)	**< 0.001**
NX	1.119 (1.006–1.245)	0.039		
With M1a				
No	1.00 (Reference)		1.00 (Reference)	
Yes	0.962 (0.848–1.091)	0.549	0.938 (0.822–1.069)	0.338
Distant metastasis				
Liver	1.00 (Reference)		1.00 (Reference)	
Bone	1.005 (0.931–1.085)	0.896	1.011 (0.934–1.094)	0.793
Brain	1.119 (1.028–1.218)	0.009	1.125 (1.031–1.228)	0.008
Radiotherapy				
Yes	1.00 (Reference)		1.00 (Reference)	
No	1.114 (1.055–1.176)	**< 0.001**	1.130 (1.068–1.194)	**< 0.001**
Unknown	1.151 (0.909–1.457)	0.244	1.132 (0.885–1.449)	0.324

M1a: Separate tumor nodules in a contralateral lobe or tumor with pleural nodules or malignant pleural dissemination; Bold values corresponds to the comparisons with $P < 0.001$.

Supplementary Table S6: Multivariate analysis of overall survival and lung cancer-specific survival in small cell lung cancer patients with single site of distant metastasis

Features	Overall survival		Lung cancer-specific survival	
	Hazard Ratios (95% CI)	*P* Value	Hazard Ratios (95% CI)	*P* Value
Age				
≤ 65 y	1.00 (Reference)		1.00 (Reference)	0.161
> 65 y	< 0.001	0.149	< 0.001	
Gender				
Female	1.00 (Reference)		1.00 (Reference)	
Male	1.094 (1.018–1.175)	0.014	1.074 (0.998–1.156)	0.058
Married				
No	1.00 (Reference)		1.00 (Reference)	
Yes	1.003 (0.932–1.079)	0.937	1.004 (0.931–1/082)	0.928
Unknown	1.009 (0.837–1.216)	0.927	1.018 (0.840–1.234)	0.856
Race				
White	1.00 (Reference)		1.00 (Reference)	
Black	1.003 (0.889–1.131)	0.966	0.996 (0.880–1.128)	0.953
Other	0.759(0.626–922)	**< 0.001**	0.679(0.550–0.838)	**< 0.001**
Location				
Main bronchus	1.00 (Reference)		1.00 (Reference)	
Single Lobe	0.969 (0.864–1.086)	0.588	0.966 (0.858–1.086)	0.560
Overlap	1.015 (0.743–1.386)	0.925	0.951 (0.684–1.322)	0.766
Unknown	1.088 (0.953–1.243)	0.213	1.090 (0.950–1.250)	0.218
T status				
T1	1.00 (Reference)		1.00 (Reference)	
T2	1.147 (1.003–1.313)	0.046	1.138 (0.990–1.308)	0.068
T3	1.209 (1.052–1.390)	0.008	1.212 (1.050–1.399)	0.009
T4	1.172 (1.028–1.335)	0.018	1.159 (1.012–1.326)	0.033
TX	1.121 (0.973–1.291)	0.114	1.142 (0.987–1.320)	0.074
Lymph node status				
N0	1.00 (Reference)		1.00 (Reference)	
N1	1.018 (0.867–1.195)	0.829	0.995 (0.843–1.173)	0.949
N2	1.099 (0.990–1.220)	0.076	1.069 (0.961–1.190)	0.220
N3	1.112 (0.981–1.261)	0.096	1.099 (0.967–1.250)	0.147
NX	1.101 (0.933–1.299)	0.254	1.069 (0.902–1.266)	0.443
With M1a				
No	1.00 (Reference)		1.00 (Reference)	
Yes	0.871 (0.689–1.099)	0.245	0.840 (0.658–1.074)	0.856
Distant metastasis				
Liver	1.00 (Reference)		1.00 (Reference)	
Bone	0.984 (0.901–1.075)	0.726	0.978 (0.893–1.071)	0.627
Brain	1.072 (0.973–1.182)	0.158	1.067 (0.966–1.180)	0.202
Radiotherapy				
Yes	1.00 (Reference)		1.00 (Reference)	
No	1.055 (0.967–1.151)	0.228	1.067 (0.975–1.167)	0.046
Unknown	0.931 (0.702–1.099)	0.618	0.951 (0.713–1.269)	0.732

M1a: Separate tumor nodules in a contralateral lobe or tumor with pleural nodules or malignant pleural dissemination; Bold values corresponds to the comparisons with $P < 0.001$.

Supplementary Table S7: Multivariate analysis of overall survival and lung cancer-specific survival in adenocarcinoma patients with multiple sites of distant metastasis

Features	Overall survival		Lung cancer-specific survival	
	**Hazard Ratios (95% CI)**	***P* Value**	**Hazard Ratios (95% CI)**	***P* Value**
Age				
≤ 65 y	1.00 (Reference)		1.00 (Reference)	
> 65 y	1.347 (1.252–1.450)	**< 0.001**	1.347 (1.249–1.452)	**< 0.001**
Gender				
Female	1.00 (Reference)		1.00 (Reference)	
Male	1.254 (1.166–1.349)	**< 0.001**	1.248 (1.158–1.344)	**< 0.001**
Married				
No	1.00 (Reference)		1.00 (Reference)	
Yes	0.809 (0.749–0.872)	**< 0.001**	0.809 (0.749–0.872)	**< 0.001**
Unknown	1.077 (0.901–1.287)	0.413		
Race				
White	1.00 (Reference)		1.00 (Reference)	
Black	1.051 (0.941–1.175)	0.375	1.028 (0.917–1.153)	0.633
Other	0.634 (0.560–0.719)	**< 0.001**	0.636 (0.560–0.722)	**< 0.001**
Location				
Main bronchus	1.00 (Reference)		1.00 (Reference)	
Single Lobe	0.814 (0.683–0.971)	0.022	0.836 (0.697–1.003)	0.054
Overlap	0.763 (0.518–1.124)	0.172	0.745 (0.497–1.118)	0.156
Unknown	0.993 (0.820–1.202)0	0.941	1.015 (0.833–1.237)	0.884
T status				
T1	1.00 (Reference)		1.00 (Reference)	
T2	1.114 (0.967–1.283)	0.134	1.148 (0.992–1.327)	0.064
T3	1.105 (0.960–1.273)	0.163	1.132 (0.979–1.309)	0.093
T4	1.170 (1.023–1.338)	0.022	1.193 (1.038–1.370)	0.013
TX	1.222 (1.045–1.429)	0.012	1.261 (1.074–1.481)	0.005
Lymph node status				
N0	1.00 (Reference)		1.00 (Reference)	
N1	0.929 (0.786–1.098)	0.388	0.919 (0.774–1.091)	0.333
N2	1.066 (0.959–1.185)	0.235	1.060 (0.951–1.180)	0.292
N3	1.054 (0.936–1.187)	0.384	1.045 (0.925–1.180)	0.481
NX	1.151 (0.972–1.363)	0.102	1.117 (0.938–1.329)	0.213
With M1a				
No	1.00 (Reference)		1.00 (Reference)	
Yes	0.801 (0.626–1.025)	0.078	0.806 (0.626–1.037)	0.094
Distant metastasis				
With Liver	1.00 (Reference)		1.00 (Reference)	
Without Liver	0.877 (0.810–0.949)	0.001	0.876 (0.808–0.950)	0.001
Radiotherapy				
No	1.00 (Reference)		1.00 (Reference)	
Yes	0.793 (0.733–0.859)	**< 0.001**	0.804 (0.741–0.872)	**< 0.001**
Unknown	1.176 (0.782–1.767)	0.436	1.214 (0.801–1.841)	0.361

M1a: Separate tumor nodules in a contralateral lobe or tumor with pleural nodules or malignant pleural dissemination; Bold values corresponds to the comparisons with $P < 0.001$.

Supplementary Table S8: Multivariatesis of overall survival and lung cancer-specific survival in small cell lung cancer patients with multiple sites of distant metastasis

Features	Overall survival		Lung cancer-specific survival	
	Hazard Ratios (95% CI)	*P* Value	Hazard Ratios (95% CI)	*P* Value
Age				
≤ 65 y	1.00 (Reference)		1.00 (Reference)	
> 65 y	1.267 (1.152–1.394)	**< 0.001**	1.260 (1.142–1.389)	**< 0.001**
Gender				
Female	1.00 (Reference)		1.00 (Reference)	
Male	1.063 (0.966–1.169)	0.209	1.046 (0.948–1.153)	0.369
Married				
No	1.00 (Reference)		1.00 (Reference)	
Yes	0.809 (0.749–0.872)	**< 0.001**	0.872 (0.789–0.964)	0.007
Unknown	0.981 (0.749–1.284)	0.889	1.009 (0.767–1.328)	0.948
Race				
White	1.00 (Reference)		1.00 (Reference)	
Black	1.079 (0.903–1.290)	0.400	1.089 (0.907–1.307)	0.361
Other	0.634 (0.560–0.719)	**< 0.001**	0.636 (0.560–0.722)	**< 0.001**
Location				
Main bronchus	1.00 (Reference)		1.00 (Reference)	
Single Lobe	1.140 (0.977–1.330)	0.096	1.141 (0.974–1.336)	0.102
Overlap	0.974 (0.665–1.427)	0.894	0.956 (0.645–1.418)	0.823
Unknown	1.217 (1.015–1.459)	0.034	1.181 (0.980–1.423)	0.081
T status				
T1	1.00 (Reference)		1.00 (Reference)	
T2	1.088 (0.898–1.319)	0.389	1.087 (0.892–1.323)	0.409
T3	1.236 (1.015–1.505)	0.035	1.216 (0.993–1.489)	0.058
T4	1.229 (1.025–1.475)	0.026	1.225 (1.017–1.477)	0.033
TX	1.246 (1.012–1.535)	0.038	1.265 (1.022–1.566)	0.031
Lymph node status				
N0	1.00 (Reference)		1.00 (Reference)	
N1	1.055 (0.831–1.339)	0.658	1.098 (0.859–1.404)	0.456
N2	1.043 (0.883–1.233)	0.617	1.074 (0.903–1.277)	0.420
N3	1.047 (0.873–1.256)	0.620	1.076 (0.891–1.300)	0.445
NX	1.325 (1.029–1.706)	0.029	1.403 (1.083–1.818)	0.010
With M1a				
No	1.00 (Reference)		1.00 (Reference)	
Yes	1.188 (0.785–1.798)	0.414	1.097 (0.704–1.709)	0.683
Distant metastasis				
With Liver	1.00 (Reference)		1.00 (Reference)	
Without Liver	0.948 (0.810–1.109)	0.505	0.876 (0.808–0.950)	0.520
Radiotherapy				
No	1.00 (Reference)		1.00 (Reference)	
Yes	0.716 (0.649–0.790)	**< 0.001**	0.720 (0.650–0.796)	**< 0.001**
Unknown	0.861 (0.572–1.294)	0.470	0.879 (0.580–1.333)	0.545

M1a: Separate tumor nodules in a contralateral lobe or tumor with pleural nodules or malignant pleural dissemination; Bold values corresponds to the comparisons with $P < 0.001$.
